# Evaluation of human obstructive sleep apnea using computational fluid dynamics

**DOI:** 10.1038/s42003-019-0668-z

**Published:** 2019-11-21

**Authors:** Shahab Taherian, Hamid Rahai, Samuel Lopez, Jamie Shin, Behrouz Jafari

**Affiliations:** 1 Center for Energy and Environmental Research and Services, California State University Long Beach, Long Beach, CA USA; 2 Department of Internal Medicine, University of California Irvine School of Medicine, Irvine, CA USA; 3 Section of Pulmonary, Critical Care, and Sleep Medicine, Veterans Affairs Long Beach Healthcare System, Long Beach, CA USA

**Keywords:** Computational models, Diagnostic markers, Translational research

## Abstract

Obstructive sleep apnea (OSA) severity might be correlated to the flow characteristics of the upper airways. We aimed to investigate the severity of OSA based on 3D models constructed from CT scans coupled with computational fluid dynamics (CFD) simulations. The CT scans of seven adult patients diagnosed with OSA were used to reconstruct the 3D models of the upper airways and CFD modeling and analyses were performed. Results from the fluid simulations were compared with the apnea-hypopnea index. Here we show a correlation between a CFD-based parameter, the adjusted pressure coefficient (Cp*), and the respective apnea-hypopnea index (Pearson’s r = 0.91, p = 0.004), which suggests that the anatomical-based model coupled with CFD could provide functional and localized information for different regions of the upper airways.

## Abstract (web summary)

Taherian et al. show a correlation between the severity of apnea-hypopnea and the adjusted pressure coefficient. This study suggests that anatomy-based models coupled with computational fluid dynamics may serve to identify biomarkers that report the severity of sleep obstruction.

## Introduction

A repetitive episode of the complete or partial collapse of the upper airway during sleep results in obstructive sleep apnea (OSA). It is a risk factor for various diseases such as hypertension, ischemic heart diseases, and diabetes mellitus^1^. Recent studies by Frost and Sullivan^2^ show that, while approximately 5.9 million adults are diagnosed with OSA in the United States, 23.5 million remained undiagnosed. The undiagnosed OSA cost is estimated to be $149.6 billion in direct economic cost, which includes, but not limited to, comorbidities such as high blood pressure, diabetes, and motor vehicle and workplace accidents. Prevalence of OSA is estimated to be 12% of the U.S adults, and 80% of this population remained undiagnosed. The comorbidities linked to the undiagnosed OSA population were approximated to be $30 billion in 2015. Furthermore, the estimated 77.4% ($86.9 billion) of the total cost burden associated with untreated OSA was due to the loss of workplace productivity^2^.

There are various biological markers (blood, exhaled breath condensate, salivary, etc) in development that may have potential value in OSA diagnosis. Despite the data in these studies, the use of inflammatory factors and cardiovascular diseases as diagnostic markers for sleep disorders remains challenging. An example of an inflammatory factor biomarker is the C-reactive protein, which can provide information for systemic inflammation; however, OSA is often a comorbid condition in which the separation of the inflammatory biomarker due to OSA alone is very challenging and often demonstrated to be elevated owing to other inflammatory conditions. A large body of research correlates OSA as an independent risk factor for cardiovascular diseases. Since a large segment of patients with OSA has hypertension, obesity, and diabetes, which independently increase cardiac risk, a cardiovascular-based biomarker may not provide specificity as an OSA biomarker^3^.

The current gold standard for diagnosing OSA is overnight polysomnography, where the numbers of apneas or hypopneas are recorded throughout the night. If the apnea–hypopnea index (AHI) value is ≥5, then the patient is diagnosed with OSA. There are many factors that can affect the AHI value. The anatomical characteristic of the upper airway, which is patient specific, plays a major role in decreasing the luminal pressure distribution and eventual collapse of the airway. Patients with OSA were found to have higher resistance in the pharynx, and increased pressure drop from the choanae to a minimum cross-section where tonsils and adenoids constrict the pharynx^4,5^.

**Fig. 1:**
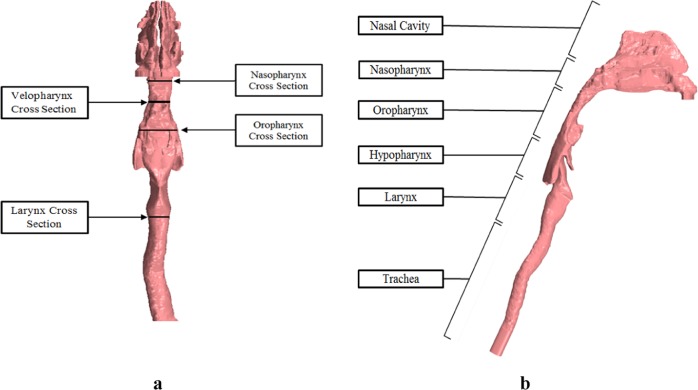
Diagram of Patient 1, CT-based model. The 3D model generated from CT images. The cross-sectional plane in which pressure and velocity magnitude values are measured are demonstrated in the frontal view (**a**). Upper airway regions are demonstrated in the sagittal (**b**) view

The respiratory system is a complex system with changing wall boundaries, rough surfaces, and moving mucus layers. The respiratory tract is divided into upper and lower airways. The upper airway includes the nasal cavity, pharynx (which includes the nasopharynx, oropharynx, velopharynx, and laryngopharynx), and the larynx, (Fig. 1b) while the lower airway includes the trachea, primary bronchi, and lungs. For a typical patient with OSA, when the pharynx muscles are relaxed, the pharynx becomes a collapsible conduit. When the pharynx is hypotonic and highly compliant, the luminal pressure decreases, resulting in reduced cross-sectional area of the segments. The most susceptible area is the velopharynx, positioned behind the soft palate, and oropharynx, which combined with aerodynamic forces can play an important role in the pathogenesis of OSA. During sleep, the narrowing process eventually results in a collapse^6–8^.

The treatment options for OSA are continuous positive airway pressure (CPAP); Mandibular Advancement Splint, which is a mouthpiece worn between upper and lower teeth when sleeping; and surgery^9^. There are various surgical treatments such as airway enlargement to keep the airways from collapsing, but these types of treatments are site dependent where inaccurate identification could result in suboptimal outcomes^1^.

Other parameters in the upper airways that could contribute to airway obstruction and correlate with the severity of OSA include: cross-sectional changes due to length and thickness of the tongue (which play a major role in upper airway obstruction^10^), hyoid bone position, oropharyngeal region, and hypopharyngeal cross-sectional area (involving the soft palate and the posterior pharyngeal wall). As the air flows between the choanae and hypopharynx, the oropharynx (retropalatal and retrolingual regions) can partially collapse and subsequently cause snoring. These regions do not have a bony or cartilaginous structure, which makes them prone to complete collapse, causing the OSA^11^.

Airway patency failure in sleep apnea patients is the result of a multitude of factors; from an aerodynamic point of view, the narrowing of the airway causes airflow recirculation and creates negative intraluminal pressure in anatomically predisposed airway regions and may cause airway collapse.

For the past decade, there has been a large body of research covering computational fluid dynamics (CFD) simulations of the upper airways. These investigations consist of CFD and fluid structural interactions in OSA and are categorized into the evaluation of pre-/post-interventions and assessment of the disease^9,12–18^. These studies and many others show the potential for CFD adaptation in predicting clinical intervention outcomes by quantification of flow and pressure changes caused by narrowing of the airway lumen. The majority of CFD-based studies correlated the response of an intervention or treatment to the percentage change of AHI. However, few investigations, including Wootton et al.^16,18^, correlated a CFD-based parameter to the severity of OSA without analyzing pre- and post-interventions. In this study, CFD modeling derived from computed tomography (CT)-based models of the upper airways was used to quantify the relationship between flow-based parameters and clinical severity of OSA measured by AHI.

## Results

**Fig. 2:**
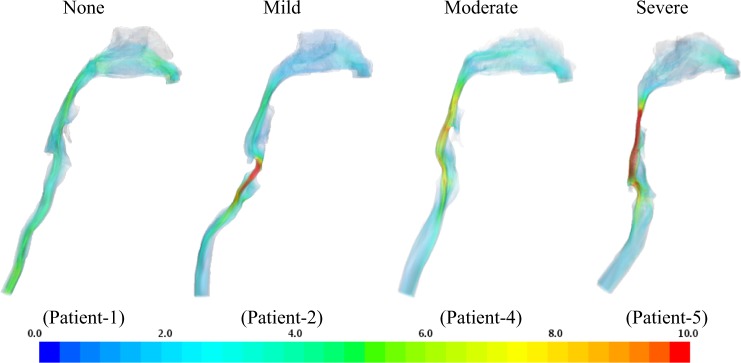
Velocity magnitude (m s^−1^). As disease severity increases, higher velocity magnitude, at 1.25 s, is observed in the oropharynx and velopharynx regions

**Fig. 3:**
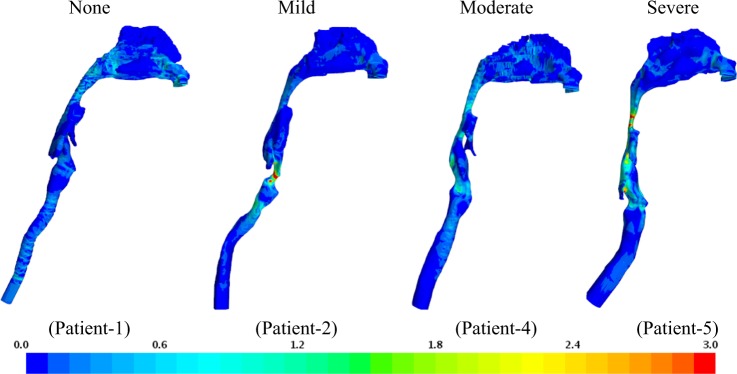
Wall shear stress (Pa). As disease severity increases, higher wall shear stress (WSS) magnitude, at 1.25 s, is observed in the oropharynx and velopharynx regions. The larynx regions experience high WSS magnitude

**Fig. 4:**
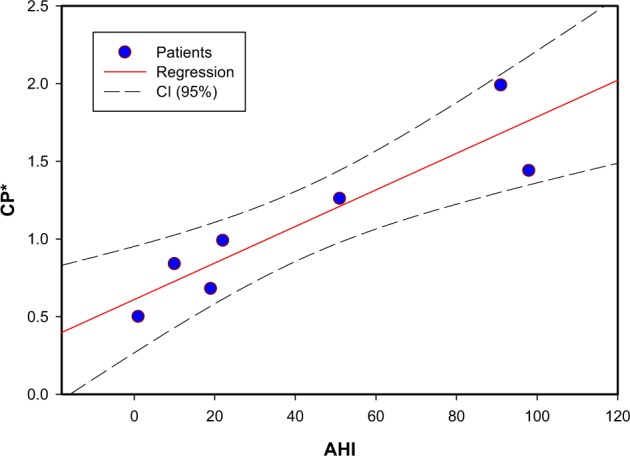
Correlation between CFD-based parameter and the apnea–hypopnea index. The analysis was two tailed and *p* < 0.01 was considered statistically significant. Pearson correlation was used to measure the degree of association between AHI, as the OSA severity (*n* = 7 patients), and the CFD parameter (Cp*). (*r* = 0.91, *p* = 0.004)

Key quantities in this investigation are the pressure on the pharyngeal wall and the velocity at the narrowing airway lumen regions. We first describe the qualitative flow behavior in a sample of a healthy, mild, moderate, and a severe OSA patient (Figs. 2 and 3) at the peak flow rate to evaluate flow characteristic variations and then provide statistical results for a total of seven patients (Fig. 4). The increase in the narrowing of the airways affects the flow pattern downstream and increases the velocity magnitude. The narrowing in the pharynx region in patient 5 creates jet flow structures; jet flow is also observed posterior of the trachea and downstream of these regions in other patients diagnosed with severe OSA (patients 6 and 7).

Regions of high recirculation are experienced downstream of the narrowing segments in patients 5 and 6 simultaneously creating a Coanda-type wall attachment similar to the flow downstream of a stenosis region. The laryngeal jet and its effect have been observed and evaluated in other investigations^19–21^. In general, regions of obstruction or constriction will experience higher levels of wall shear stress (WSS). Figure 3 shows WSS distributions in four patients. High WSS is observed for patient 1 in the nasopharynx region, which reduces downstream until it becomes nearly uniform in the trachea region. Higher WSS is observed in the oropharynx and hypopharynx regions for patient 4. The highest WSS is observed in patients 5 and 6 along the entire pharynx region, especially at the oropharynx region. As observed in previous investigations, the laryngeal region generally experiences relatively high WSS for all models. In the cases of patients with mild and severe OSA, an increase in WSS is observed at the constriction, indicating an increase in velocity gradient and a jet-like flow structure. Similar behavior has been observed in patients with moderate OSA with less magnitude. However, this shows that WSS and AHI are weakly correlated.

The current study introduces a relationship between the adjusted pressure coefficient (Cp*) and AHI as a parameter for CFD evaluation of the disease. Cp* is herein described as the square root of the ratio of pressure changes to the velocity squared.

1 $${\mathrm{Cp}}^{\ast} = \sqrt {\frac{{\left| {P_{\rm{o}} - P_{\rm{n}}} \right|}}{{V_{\rm{v}}^{2}}}}$$

The pressure changes used for this study are at the nasopharynx (*P*_n_) and oropharynx cross-sections (*P*_o_), while the averaged velocity magnitude is measured at the velopharynx cross-section (*V*_v_) (Fig. 1a).

Figure 4 demonstrates the correlation (*r* = 0.91, *p* = 0.004) between patient’s AHI values and Cp* from CFD investigations. It should be noted that oropharynx and velopharynx regions are included between nasopharynx and oropharynx cross-sections (Fig. 1), since in most individuals this area is considered to be the most collapsible part of the upper always. These regions along with their strong pressure gradients are important upper airway structures for consideration of OSA evaluation^6,13,22^. Thus pressure measurements were at nasopharynx and oropharynx cross-sections, and velocity measurements were at the narrowest area, velopharynx cross-section.

The findings generally indicate that the increase in WSS, pressure drop, and velocity magnitude induced by narrowing of the airway lumen are experienced in higher degree as the AHI scoring increases. However, this is not true for all patients. Patient 7 highlights this matter; the velocity, pressure, and WSS magnitudes alone do not provide a strong correlation with OSA severity; for example, pressure drop at velopharynx and oropharynx cross-section was weakly correlated to AHI and did not reach statistical significance. Cp* provides a better correlation with AHI value.

It should be noted that, although patient 7 has a higher AHI value (98 per hour) than patient 6 (91 per hour), the CFD index (Cp*) shows a lower value. This may be due to the airway compliance, which requires analysis based on inspiratory–expiratory CT-based models, and a larger number of patients with similar AHI values.

## Discussion

The underlying pathophysiology of OSA, due to a complex and heterogeneous nature of the disease, is not well understood^23^. Although the varying levels of anatomical narrowing (static or dynamic) is a very important factor, other non-anatomical features (such as arousal threshold, loop gain, and muscle responsiveness) are contributors to this syndrome, resulting in different phenotypes^24^.

The upper airway is a complex area with a different outline, muscle groups, and responsiveness, but the final product in OSA is partial or complete obstruction. We used image-based CFD to estimate the upper airway pressure changes at several locations. Functional evaluation of the upper airway using CFD assess the flow of the air in a manner similar to that during actual breathing, even in cases of upper airways with complicated morphologies and may predict pharyngeal-airway collapse during sleep^16,25^.

In the present study, we focused on oropharynx and velopharynx regions due to the higher probability of collapse to evaluate the airflow characteristics and correlate a CFD parameter with OSA severity. Our findings, in contrast with others, indicated that the increased air velocity, WSS, and pressure drop in the narrowed area alone do not provide a strong association with OSA severity, but the adjusted pressure coefficient (Cp*) provides a better correlation with AHI value, which is a new finding for the first time. The Cp* describes the relative pressures throughout a flow field. The pressure coefficient, in a three-dimensional (3D) model can be determined at critical locations and can be used with confidence to predict the air or fluid pressure changes at those locations.

We found that the Cp*, as a parameter for CFD evaluation of the disease, renders an individual relationship with air velocity and pressure drops in the upper airway and it is strongly correlated with the AHI values (*r* = 0.91, *p* = 0.004). The high correlation could indicate that this area is more susceptible to collapse, resulting in a more severe condition.

Previous investigations found that effective compliance did not correlate strongly to pharyngeal pressure drop as well, which raises the possibility that effective compliance is independent of pressure drop^18^. Other results indicate that the nasopharynx (in contrast to ours, velopharynx) is more critical since it narrows the airway leading to the velopharynx^16,18^; however, the study also alternatively suggests that, in obese children with OSA, with a different phenotype, their pharyngeal dilators are less effective in controlling the airway lumen cross-section at the velopharynx, thus leading to more expansion at the nasopharynx. This indicates that using an image-based CFD approach can help us better understand the underlying mechanism and different phenotypes.

Our finding is also more consistent with Zhao et al.^9^, which found no correlation between the airway geometry and AHI; however, they found that changes in the square root of the pharyngeal pressure drop ($\Delta \sqrt {\Delta P_{{\rm{max}}}} \%$) was strongly correlated (*r* = 0.97, *p* = 0.00016) to AHI.

Image-based CFD analysis of upper airways can be a potential tool to differentiate subjects with and without OSA. Cisonni et al.^6^ introduced a flow-based criterion, as a ratio between velopharyngeal and total pharyngeal pressure drops. Their results demonstrated that small geometrical differences in the narrowed airway could lead to variations of intraluminal pressure characteristics among subjects. The clearest criterion to distinguish the two groups was the average pressure drop in the velopharynx area, which was more consistent with our findings. The main consequence of this high-pressure drop was the existence of a low-pressure field downstream of the velopharynx, hence the susceptibility of the airways to collapse. The upper airway collapse in an OSA patient at the oropharynx, with low oropharyngeal pressure induced by velopharyngeal jet flow, was also observed by Zhao et al.^22^.

Our technique can potentially be used to differentiate normal vs OSA subjects; it can also be considered as a mean to measure the effectiveness of treatment outcomes. Luo et al.^14^ investigated the correlation between adenotonsillectomy treatment response and variations in airway geometry, maximum pressure drop, and pressure–flow ratio, among other parameters. They found that AHI decreases after the treatment and correlates with the reduction of maximum pressure drop (*r* = 0.78, *p* = 0.011) in the tonsils and adenoid regions where the pharynx is narrowed. The correlation suggests that maximum pressure drop might be a useful index for internal airway loading due to anatomical narrowing and maybe better correlated with AHI than direct airway anatomic measurements.

In another study, Lu et al.^15^ observed in two patients’ pre- and post-surgical interventions that the location of the largest aerodynamic force distribution is important, since a strong negative pressure can cause airway collapse and a large WSS may lead to eventual injury to the wall of the airways.

Normalization of both the CFD results and AHI by patient-specific values measured prior to intervention have been observed in most studies. Although these studies support the idea of using CFD as a predictive mechanism for clinical intervention outcomes, there is a need to correlate anatomically based CFD results to the severity of the disease.

The anatomically based studies show potential for CFD as a predictor for clinical interventions and disease evaluation. However, there are limitations to CFD-based disease and pre-/post-intervention evaluations. The assumption of rigid wall boundary conditions, awake medical imaging, and pure nasal breathing are the main limitations of this study. The rigid wall boundary condition in awake medical imaging and normal breathing cycle provide reasonable accuracy with regards to airway simulation; however, this means that muscle and structural tension variation, especially in sleep conditions, are not considered. This might be the reason for the interpatient variability of CPAP pressure values for patients with similar AHI, age, and BMI.

Assessment of airway compliance is an important parameter that has not been modeled in this study. Automated volume preservation in addition to manual editing and smoothing, during 3D model reconstruction from CT images, were used to minimize the effects of noise and stair-step artifacts due to coarse axial image spacing; however, these necessary steps might reduce the resolution of realistic anatomical models. The assumption of nasal breathing can also be limiting since, during sleep, nasal breathing can switch to oral breathing due to nasal obstruction or high nasal resistance.

Although the CFD approach to the upper airway physical properties has the potential for better understanding of the different phenotypes and personalized medicine for those group of patients with upper airway impairment, imaging cannot be considered a screening tool at this point, and further prospective investigation with a larger number of patients is required for better assessment^26,27^. The evaluation of these parameters and their respective sensitivities, interpatient variability, and inclusion of world data and not just US-based data in the CFD study are the authors’ ongoing investigation.

## Methods

### CT-based model

A total of seven subjects with CT images and the associated overnight polysomnography studies were evaluated (Table 1). The associated protocol was approved by the VA Long Beach Healthcare System IRB committee.

**Table 1:** Patient classification

	Patient 1	Patient 2	Patient 3	Patient 4	Patient 5	Patient 6	Patient 7
AHI classification	1	10	19	22	51	91	98
	None	Mild	Moderate	Moderate	Severe	Severe	Severe

**Fig. 5:**
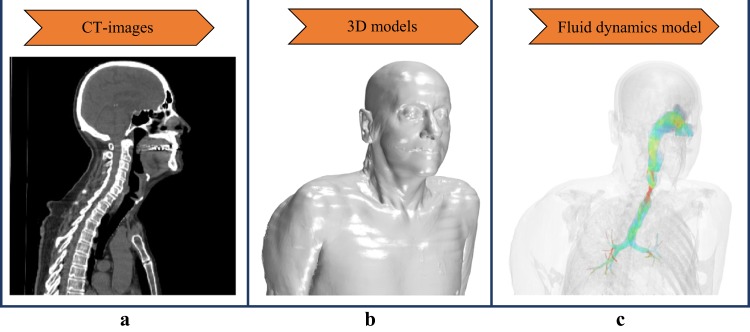
Workflow. Realistic anatomical models and their respective CFD results can be evaluated using the following workflow: CT images of patients are collected (**a**); using manual and automated segmentation tools, 3D models are generated (**b**); and flow characteristic and flow-based parameters are calculated using CFD simulations (**c**)

In this investigation, the CT images of a healthy subject and patients with mild, moderate, and severe sleep apnea were taken during awake respiration. The acquired images in Digital Imaging and Communications in Medicine (DICOM) format were converted into 3D models using the commercial software Mimics (Materialise Leuven, Belgium). The airways were segmented from axial images; the image intensity threshold set was based on an initial selection of pixels with Hounsfield units ranging between −1024 and −350. To compensate for variations in image noise, locally poor anatomical resolution and artifacts, automated and manual surface smoothing as well as volume-preserving tools were used to create a realistic model without loss of patient-specific morphology (Fig. 5b).

Patient-specific airway geometry affects the flow behavior; thus the inclusion of appropriate regions such as the nasopharynx, oropharynx, and larynx are essential for upper airway simulations (Fig. 1).

The velopharyngeal region is combined with oropharynx to simplify the selection of the regions across patients. Owing to variations in flow characteristics in the trachea, the idealization of these regions is impractical. As the flow reaches downstream to deeper generations of the airway, the effect of the upper regions becomes minimal, thus reducing the need to include the branching airways for upper airway analysis^28^.

The flow passing through the nasal passages become turbulent before entering the pharynx region. Complete exclusion of the nasal cavity may result in unrealistic characteristics of the velocity flow and pressure fields. However, an artificial inlet extension may compensate for these effects. Cisonni et al.^12^ have investigated the effects of various alternatives for the nasal cavity; they have concluded that, by simplifying the nasal cavity to a convergence channel, the flow condition upstream of the pharynx can be reproduced with high precision. Wootton et al.^16^ have also suggested that modeling of the pharynx alone may be sufficient for OSA analysis due to the minimal impact of the nasal cavity. This matter has also been investigated by Taherian et al.^28^ where artificial inlet extensions were added at the beginning of the nasopharynx regions, thus reducing the computational cost and 3D modeling complexity. Simplification of this region can only be considered if a deviated septum and/or other obstructions are not present in the nasal cavity. However, to minimize the uncertainty, the nasal cavity was included in this study.

### CFD set-up

The completed models were imported into the STAR-CCM+ (Siemens) software for CFD analyses (Fig. 5c). Inlet and outlet boundary conditions were assigned. A mass flow outlet boundary condition was assigned at the outlet to simulate normal resting respiration (tidal volume) and was consistent across all patients to highlight the effect of the anatomically based model on the results.

The implicit unsteady shear stress transport *k*-*ω* turbulence model was used with low Reynolds number modification to model the flow turbulence in normal respiration, as previously described^29,30^. The CFD results for the first patient, excluding the nasal cavity, were correlated with the corresponding experimental data from a light-emitting diode–particle image velocimetry system^31^. The numerical investigations included large eddy simulations and Reynolds-Averaged Navier–Stokes equations with *k*-*ε* and *k*-*ω* turbulence models. The large eddy simulation approach provided more details of the velocity field around obstruction with velocity gradients and recirculation. Results were compared with the corresponding experimental data; while the numerical and experimental results agreed qualitatively, the experimental results lacked the details that were displayed in the numerical results due to seeding limitations. In general, the experimental velocity values were less than the corresponding numerical values.

A sinusoidal wave function:

2 $$V_{t} = A_{t}\sin \left( {\frac{{2\pi }}{\tau }( t)} \right)$$

that shows the volumetric rate of respiration *V*_*t*_ was defined to emulate the mass flow at the outlet for realistic breathing conditions. *A*_*t*_ is related to the minute volume of inspiration, the product of tidal volume and the number of breathings per minute. *τ* is the cycle time of a single breath and *t* being the corresponding time step; mass flow rate was assigned using sinusoidal-type flow behavior during normal tidal volume (500 ml) for a single breath. The simulations were performed for 2.5 s of physical time, with characteristic time steps of ∆*t* = 10^−3^ s, using a second-order implicit unsteady scheme. A turbulence intensity of 10% and no-slip boundary condition were assumed at the inlet.

### Mesh

Polyhedral and prism layer meshes were used to capture the computational domain of each model. The wall unit (*y*+) represents the dimensionless normal distance from the wall (or surface of the airway) to the nearest mesh cell in the viscous sub-layer. The *y*+ value was <1 throughout the entire domain for all subjects, which was sufficient to resolve the near wall flow dynamics. The mesh independency analysis was performed at 1.25 s (at the peak of the inhalation). The pressure drop variations in the healthy subject for 0.9, 3.2, and 12 million mesh cells were compared with 6.4 million cells. The length scale used to generate the 12 million mesh cells was the same as Taylor microscale. The results changed by 4.5%, 1.2%, and 0.5%, respectively, thus the 6.4 million cell sizes were adopted for the rest of the models.

### Statistics and reproducibility

The analysis was performed using the SPSS software package. The analysis was two tailed and *p* < 0.01 was considered statistically significant. Pearson correlation was calculated to test the association between AHI, as the OSA severity, and the CFD parameters.

### Reporting summary

Further information on research design is available in the Nature Research Reporting Summary linked to this article.

## Supplementary information

Description of Additional Supplementary Files

Supplementary Data 1

Reporting Summary

## Data Availability

The data that support the findings of this study are available from the corresponding author upon reasonable request. The source data underlying Fig. 4 are shown in Supplementary Data 1.
