# Supplementary material for: Evaluation of human obstructive sleep apnea using computational fluid dynamics
Source: Commun Biol. 2019 Nov 21;2:423. doi: 10.1038/s42003-019-0668-z (PMC6872714; doi:10.1038/s42003-019-0668-z)
Supplement: Supplementary file 1 — Description of Additional Supplementary Files [file 42003_2019_668_MOESM1_ESM.pdf]

Description of Additional Supplementary Files

File Name: **Supplementary Data 1**

Description: The analysis was two-tailed and $p<0.01$ was considered statistically significant. The Pearson correlation was used to measure the degree of association between AHI, as the OSA severity, ($n=7$ patients) and the CFD parameter(C_p*). ($r=0.91$, $p=0.004$)
